# Foraging by forest ants under experimental climatic warming: a test at two sites

**DOI:** 10.1002/ece3.473

**Published:** 2013-01-18

**Authors:** Katharine L Stuble, Shannon L Pelini, Sarah E Diamond, David A Fowler, Robert R Dunn, Nathan J Sanders

**Affiliations:** 1 Department of Ecology and Evolutionary Biology, University of TennesseeKnoxville, Tennessee, 37996; 2 Department of Biological Sciences, Bowling Green State UniversityBowling Green, Ohio, 43403; 3 Department of Biology, North Carolina State UniversityRaleigh, North Carolina, 27695

**Keywords:** Climate change, critical thermal maximum, foraging, thermal tolerance, warming

## Abstract

Climatic warming is altering the behavior of individuals and the composition of communities. However, recent studies have shown that the impact of warming on ectotherms varies geographically: species at warmer sites where environmental temperatures are closer to their upper critical thermal limits are more likely to be negatively impacted by warming than are species inhabiting relatively cooler sites. We used a large-scale experimental temperature manipulation to warm intact forest ant assemblages in the field and examine the impacts of chronic warming on foraging at a southern (North Carolina) and northern (Massachusetts) site in eastern North America. We examined the influence of temperature on the abundance and recruitment of foragers as well as the number of different species observed foraging. Finally, we examined the relationship between the mean temperature at which a species was found foraging and the critical thermal maximum temperature of that species, relating functional traits to behavior. We found that forager abundance and richness were related to the experimental increase in temperature at the southern site, but not the northern site. Additionally, individual species responded differently to temperature: some species foraged more under warmer conditions, whereas others foraged less. Importantly, these species-specific responses were related to functional traits of species (at least at the Duke Forest site). Species with higher critical thermal maxima had greater forager densities at higher temperatures than did species with lower critical thermal maxima. Our results indicate that while climatic warming may alter patterns of foraging activity in predictable ways, these shifts vary among species and between sites. More southerly sites and species with lower critical thermal maxima are likely to be at greater risk to ongoing climatic warming.

## Introduction

Climatic warming is currently shifting the phenologies and ranges of species (Parmesan and Yohe 2003; Chen et al. 2011), as well as relative abundances of species within communities (Walker et al. 2006). Warming may also alter behavior of individuals in those communities (Kearney et al. 2009; Walther 2010; Kordas et al. 2011). However, variation in the extent to which increasing temperatures alter rates of foraging likely exists among species and ecosystems. To a large degree, the vulnerability of a species to warming is mediated by the difference between thermal limits of, and temperatures experienced by, individuals (Kingsolver 2009; Huey et al. 2012). A change in the thermal regime is more likely to affect individuals that have smaller differences between thermal limits and environmental temperatures. This proximity to the critical thermal maximum is driven both by the thermal tolerance of the individuals within a species as well as the thermal regime of the environment, making both species identity and geographic location (or background climate) important components in predicting responses to climatic warming (Kingsolver 2009). All things being equal, this suggests that a larger effect of warming might be expected where conditions are already warm and individuals occur closer to their critical thermal maxima (CT_max_) (Deutsch et al. 2008). For example, tropical species typically occur at temperatures that are closer to their critical thermal maxima than do temperate species, making them more susceptible to the detrimental effects of warming (Deutsch et al. 2008; Tewksbury et al. 2008; Kingsolver 2009; Diamond et al. 2012a; Huey et al. 2012). While the differences between the responses of tropical and temperate ectotherms to climate warming are apparent, the question of whether this pattern holds at higher and lower latitudes within the temperate zone is less clear.

Ants are ubiquitous in most terrestrial ecosystems and interact broadly with other species. As a consequence, changes in ant activity, as well as local and regional distributions (including those caused by temperature), can produce changes in ecosystem function, including nutrient transport and seed dispersal, among other impacts (Wardle et al. 2011; Zelikova et al. 2011). Given the influence of temperature on ants, and the often substantial roles, ants play in ecosystems, any change in temperature that affects ants could have cascading effects throughout terrestrial ecosystems.

In this study, we experimentally warmed ant assemblages from ambient to 5.5°C above ambient temperatures over a period of 9 months (Pelini et al. 2011b) to examine the impact of temperature on ant foraging activity across species and populations. To our knowledge, this study is among the first field manipulations to experimentally warm intact animal assemblages, with replication at the southern and northern boundaries of an extensive geographic area. Manipulations were conducted at two distinct locations in order to assess shifts in ant activity both near the northern and southern range extents of several forest ant species in eastern North America. Such experimental warming allowed us to examine explicitly the impacts of temperature on ant foraging activity, as well as how the impacts might differ at northern and southern range boundaries and among species. Here, we tested four explicit predictions:

1. Forager abundance of individual species varies with temperature treatment (Δ°C), and the responses of species to warming depend on the maximum thermal tolerances (CT_max_) of the species.
2. Species richness at baits is lower in higher temperature (Δ°C) treatments.
3. Warming alters the ability of ants to recruit to food resources.
4. Finally, the magnitude of overall and species-specific shifts in forager abundances, as well as declines in richness, in response to increased temperatures is greater at the southern site than the northern site, because southern species operate closer to their critical upper thermal limits.

## Materials and Methods

To examine the effects of chronic warming on ant foraging, we experimentally manipulated air temperature at two sites - a southern site (Duke Forest in North Carolina, USA) and a northern site (Harvard Forest in Massachusetts, USA). The experimental site at Duke Forest (35° 52′0″N, 79°59′45″W, 130 m above sea level (a.s.l.)) is in an ∼80-year old oak-hickory stand. The mean annual temperature at Duke Forest is 15.5°C, and the mean annual precipitation is 1140 mm. The experimental site at Harvard Forest (42°31′48″N, 72°11′24″W, 300 m a.s.l.) is in a ∼70-year-old oak-maple stand in the Prospect Hill Tract. The mean annual temperature at Harvard Forest is 7.1°C, and the mean annual precipitation is 1066 mm. Despite the 8°C temperature difference, Duke Forest and Harvard Forest share more than 30 ant species (Pelini et al. 2011b). Furthermore, species found at both sites tend to be at or near their northern range limits in Massachusetts and at or near their southern range limits in North Carolina.

The temperature manipulation consists of 12 open-top chambers at each site. Each chamber is an octagon that is 5 m in diameter and 1.5 m tall. There is an approximately 3 cm gap at the bottom of each chamber which, along with the open top, allows for movement of ants in and out of the chambers. The air within these chambers is actively warmed as described in Pelini et al. (2011b) with nine chambers set to increase ambient air temperatures by approximately 1.5–5.5°C above ambient temperatures at half-degree steps, with one chamber at each temperature treatment. The three remaining chambers are controls and blow air at ambient temperatures into the chambers. Chambers have been warmed continuously since January 2010. Air temperature within the chambers is monitored continually by each of two thermisters connected to a data logger (see Pelini et al. 2011b).

Within each chamber, we placed four evenly spaced bait stations, each consisting of two resource solutions (20% sugar and 20% protein to increase the number of ant species collected). Paired sugar and protein tubes were spaced 1 m apart from one another. We deployed all resource tubes at 11am to sample at a time when the majority of species were foraging. Sampling was conducted in the summer and fall at both sites.

### Resource tubes

The 20% protein solution was made with unflavored whey protein powder (Jay Robb Enterprises, Carlsbad, CA). Both resource solutions consisted of 10-mL of solution in a 50-mL centrifuge tube containing a cotton ball to soak up the solution (Kaspari et al. 2008). Tubes were placed such that the opening was flat against the surface of the ground or leaf litter, allowing ants easy access to the resource. After 2 h (at 1 pm), the two resource tubes were capped and returned to the lab where all ants were identified to species. Liquid baits have been found to attract the same suite of species that are collected using other common bait types within the warming chambers (personal observation). Moreover, these techniques are being used widely to assess resource limitation in ant communities (Kaspari and Yanoviak 2001; Kaspari et al. 2008, 2010). As with any baiting protocol, there is some chance that interference or aggressive interactions from early discovers deter species that arrive later at the baits. However, we are not interested in quantifying competitive dominance or discovery by particular species in this particular study (but see Stuble et al. in press). Instead, we seek to document the response of the entire assemblage to resources. *Aphaenogaster rudis* and *Aphaenogaster carolinensis* were combined under the *A. rudis* complex due to their perceived morphological and ecological similarity in the field.

### Forager abundance

We calculated the number of tubes of each resource per chamber (a maximum of four) occupied by ants, as well as by each individual species, for each season and site combination. Because ants are social and live in colonies, which are the unit of selection, estimating abundance is challenging. Therefore, many investigators use occupancy as an estimate of abundance (Kaspari 2001; Longino et al. 2002; Sanders et al. 2007). Here, we use bait occupancy (number or proportion of baits occupied), which is also often used as an estimate of ant abundance (Holway 1998; Ratchford et al. 2005; Wittman et al. 2010). We used ANCOVA to examine the effects of temperature treatment (Δ°C, which is the degrees Celsius above ambient temperature), as a continuous variable as well as site (Duke Forest or Harvard Forest), whereas controlling for season, and resource type (protein or sugar) (included as fixed effects), on overall and species-specific bait occupancy. Site and/or season were removed as factors from the model for species that were entirely absent from a given site or season. Models were subsequently run separately for each site to examine the relationship between temperature treatment and forager abundance in cases in which there was a significant site-by-treatment interaction. All analyses were conducted using SAS version 9.2 and for all analyses, we tested for all combinations of interactions, sequentially removing nonsignificant interactions. We square-root transformed data on overall ant occupancy of resource tubes to meet normality assumptions (Bolker et al. 2009). For clarity, we present untransformed data in the figures and tables. Species-specific models were not run for species observed in fewer than eight resource tubes.

### Species richness

In order to collect ants that were foraging in the chambers but not necessarily visiting the resource tubes during the summer sampling period, we hand sampled for 5 min in each chamber following the baiting trial. Representatives of all species seen in these 5 min were collected. We combined these data with the data from the resource tube experiment to estimate total richness for each chamber. We again used ANCOVA to examine the effects of Δ°C (included as a continuous variable) and site (included as a categorical variable) on species richness. We square-root transformed richness data to meet the normality assumptions of ANCOVA.

### Recruitment

We estimated recruitment as the number of workers in a resource tube for each species that discovered the tube, as well as for all ant species combined. We analyzed the data using ANCOVA with Δ°C as a continuous variable, and site, season, and resource as discrete variables. Site and/or season were removed as factors from the model for species that were entirely absent from a given site or season. To meet assumptions of normality, overall recruitment data (data for all ants, combined) were cube-root transformed. For species-specific recruitment data, we log-transformed recruitment by both *Aphaenogaster rudis* and *Crematogaster lineolata* (recruitment data for the remaining species did not need to be transformed).

### Thermal tolerance

Finally, we calculated the mean temperature at which each species at Duke Forest was found foraging during both the summer and the fall. To examine the relationship between thermal tolerance and foraging activity in hot chambers, we used the critical thermal maxima (CT_max_) determined for ants at Duke Forest in the summer of 2010 based on the temperature at which locomotive coordination was lost (the temperature was raised 2°C every 10 min) (Diamond et al. 2012a). The rate of experimental warming and use of ramping experiments can affect estimation of thermal tolerance (Rezende et al. 2011; Terblanche et al. 2011), but we note that identical methods were used for all the study species included here. The mean foraging temperature for each species was regressed against the CT_max_ for each species, based on worker abundances in resource tubes. We performed separate regressions for summer and fall, as ambient temperatures differed between seasons and not all species were sampled in both seasons. Harvard Forest was not considered in this analysis because only three species were present in tubes, limiting our ability to conduct meaningful statistical analyses.

## Results

### Forager abundance

**Figure 1:**
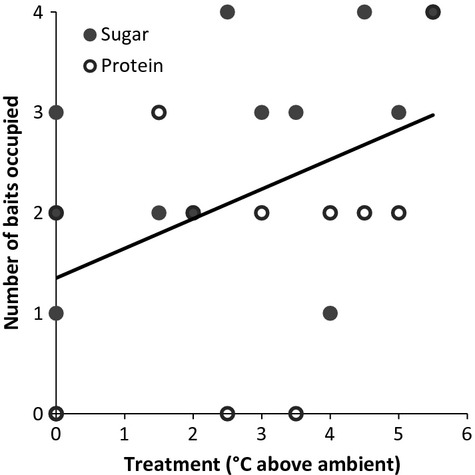
Ant occupation of protein and sugar baits as a function of temperature treatment. There is no significant effect of temperature treatment on ant abundance, although there was a significant site × treatment interaction. Only data from Duke Forest in the summer are shown, showing a positive relationship between bait occupancy and temperature treatment. The line is the best-fit line through all the points, regardless of bait type.

We observed *Aphaenogaster lamellidens*, *A. rudis*, *Camponotus pennsylvanicus*, *C. lineolata*, *Formica pallidefulva*, *Nylandaria faisonensis*, *Prenolepis imparis*, and *Temnothorax curvispinosus* in resource tubes at Duke Forest and *A. rudis*, *C. pennsylvanicus* and *Myrmica punctiventris* at Harvard Forest. Overall ant occupancy of resource tubes per chamber (as measured by the number of baits containing ants in a chamber) was 6× higher at Duke Forest (1.2 ± 0.2 baits chamber^−1^) than Harvard Forest (0.2 ± 0.1 baits chamber^−1^) and 6.7× higher in the summer (1.3 ± 0.2 baits chamber^−1^) than in the fall (0.2 ± 0.1 baits chamber^−1^). Overall, ant occupancy did not depend on Δ°C (F_1,90_ = 2.42, *P* = 0.12) (Fig. 1), but there was a significant site-by-Δ°C interaction (F_1,90_ = 3.95, *P* = 0.05) such that Δ°C and ant occupancy were positively correlated at Duke Forest (F_1,44_ = 5.72, *P* = 0.02, *R*^2^ = 0.58), but not at Harvard Forest (F_1,44_ = 0.16, *P* = 0.70).

Responses to temperature treatment varied among species. Resource tube occupancy by *C. pennsylvanicus* and *A. rudis* remained unrelated to Δ°C, whereas *P. imparis* was negatively associated with Δ°C, and *F. pallidefulva* and *C. lineolata* were positively associated with Δ°C (Table 1). Only *A. rudis* and *C. pennsylvanicus* were present at both sites in numbers great enough to allow for analysis of between-site variation, although neither species differed significantly in forager abundance between the two sites or among temperature treatments (Table 1).

**Table 1:** ANCOVA table of ant abundance as measure by the number of resource tubes containing a worker of a given species. When species were only observed during on season or at one site, season and/or site were not included as factors. Interactions were removed from the model when nonsignificant. Treatment refers to the experimental warming treatment

Species	Variable	d.f.	F	*P*
*Aphaenogaster rudis* Enzmann	Treatment	1,91	0.56	0.46
	Resource	1,91	8.32	<0.01
	Site	1,91	0.07	0.79
	Season	1,91	5.57	0.02
*Camponotus pennsylvanicus* (De Geer)	Treatment	1,91	0.02	0.87
	Resource	1,91	9.36	<0.01
	Site	1,91	2.89	0.09
	Season	1,91	9.36	<0.01
*Crematogaster lineolata* (Say)	Treatment	1,44	4.33	0.04
	Resource	1,44	0.87	0.36
	Season	1,44	24.64	<0.01
*Formica pallidefulva* Latreille	Treatment	1,21	4.50	0.05
	Resource	1,21	0.67	0.42
*Prenolepis imparis* Emery	Treatment	1,21	13.30	<0.01
	Resource	1,21	0.48	0.49

### Species richness

**Figure 2:**
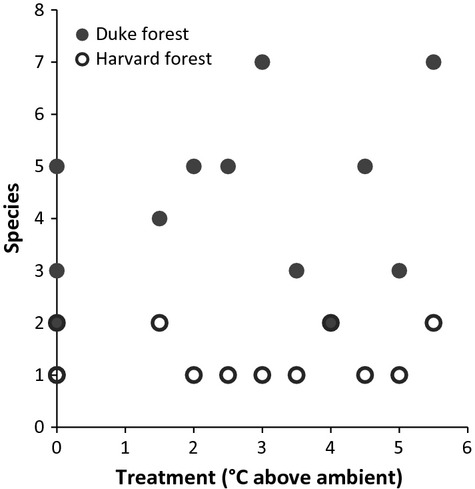
Species richness as a function of temperature treatment. There is no relationship between temperature treatment and richness at either site.

Species richness of actively foraging ants was 3.2× higher at Duke Forest (4.3 ± 0.5 species chamber^−1^) than at Harvard Forest (1.3 ± 0.5 species chamber^−1^). Temperature treatment (Δ°C) was not related to species richness (*F*_1,20_ = 1.03, *P* = 0.31) (Fig. 2), but there was a significant temperature × site interaction (*F*_1,20_ = 2.55, *P* = 0.02) such that species richness at Duke Forest was marginally positively correlated with Δ°C (*F*_1,10_ = 4.04, *P* = 0.07), whereas there was no relationship between richness and Δ°C at the Harvard Forest site (*F*_1,10_ = 2.82, *P* = 0.12).

### Recruitment

Recruitment (number of ants in a resource tube assuming that tube had been discovered) was not related to Δ°C after controlling for the effects of site, season, and resource type (total ants on baits regardless of species identity) (*F*_1,31_ = 1.51, *P* = 0.23). This was also true when considering recruitment for individual species (Table 2), which showed no response to variation in Δ°C.

**Table 2:** ANCOVA table of recruitment ability (number of workers if a species was present on a bait). Treatment refers to the experimental warming treatment

Species	Factor	d.f.	F	*P*
*Aphaenogaster rudis*	Treatment	1,11	0.76	0.40
	Resource	1,11	0.85	0.38
	Site	1,11	0.03	0.86
	Season	1,11	2.32	0.16
*Camponotus pennsylvanicus*	Treatment	1,5	1.02	0.34
	Resource	1,5	0.04	0.85
*Crematogaster lineolata*	Treatment	1,14	1.21	0.29
	Resource	1,14	4.82	0.05
	Season	1,14	6.57	0.02
*Formica pallidefulva*	Treatment	1,2	1.91	0.3
	Resource	1,2	0.13	0.75
*Prenolepis imparis*	Treatment	1,11	0.43	0.52
	Resource	1,11	17.95	<0.01

### Thermal tolerance

**Figure 3:**
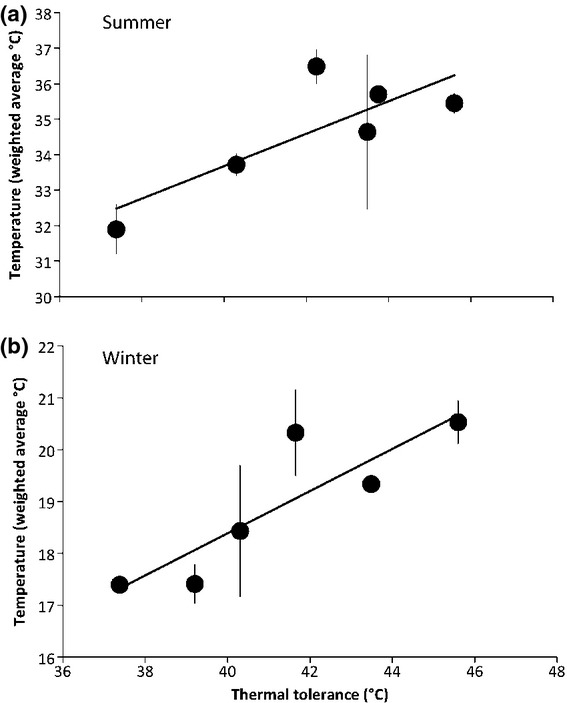
Mean temperature (±standard error) at which a species was observed foraging as a function of the critical thermal maximum in a) the summer and b) the fall at Duke Forest. Each point represents a species and the line represents the best-fit linear regression.

At Duke Forest, species with a higher CT_max_ tended to be more abundant in warmer chambers than did those species with a lower CT_max_. CT_max_ across species was significantly correlated with the mean temperature at which individuals were found foraging in both the summer (*F*_1,4_ = 7.73, *P* = 0.0498, *R*^2^ = 0.66) and fall (*F*_1,4_ = 12.76, *P* = 0.02, R^2^ = 0.76) at Duke Forest (Fig. 3). There were six species present in both the summer and fall at Duke Forest, with four species present in both seasons: *A. rudis*, *C. lineolata*, *N. faisonensis*, and *T. curvispinosus*. In the summer, *C. pennsylvanicus* and *F. pallidefulva* were also present, whereas *A. lamellidens* and *P. imparis* were present in the fall. This analysis included ants from only Duke Forest because the Harvard Forest site was too depauperate to perform meaningful analyses.

## Discussion

Climatic warming continues to change the structure and function of communities (Parmesan 1996; Thomas et al. 2006; Kardol et al. 2010; Walther 2010; Sheik et al. 2011). However, the magnitude and direction of these changes may vary by region (Kingsolver 2009; Pelini et al. 2011a) and depend on community composition (Williams et al. 2008). By experimentally manipulating temperatures of intact ant assemblages, we found evidence for shifts in foraging activity of several common generalist ant species consistent with predictions based on their thermal tolerances at the southern site. Importantly, however, the relationship between foraging activity and temperature treatment (Δ°C) varied between the northern and southern sites, with greater impacts at the more southern site.

These results are not entirely surprising as temperature clearly influences foraging in ants and many other ectotherms (Traniello et al. 1984; Cerdá et al. 1997, 1998; Ruano et al. 2000; Hurlburt et al. 2008). More basically, temperature, at least for some species, can also regulate the onset and cessation of foraging, whether daily (Talbot 1943), or seasonally (Sanders 1972; Markin et al. 1974). These factors may ultimately influence both the likelihood that an ant will be able to find food resources under warmed conditions, and also the extent to which a species will recruit to that food resource once it is discovered. However, the effects of temperature increases on foraging activity are likely driven, in part, by species-specific thermal tolerances. As such, thermal tolerance can ultimately regulate ant foraging activity in a warmer climate and determine the susceptibility of ants to climatic warming (Diamond et al. 2012a). In addition, other temperature-related factors, including the rapid breakdown of pheromones at high temperatures and running speeds, likely play a role in regulating the impacts of temperature on ant foraging activity (Hurlburt et al. 2008; van Oudenhove et al. 2011, 2012). Levels of foraging activity may also be influenced by any shifts in worker abundance caused by warming.

The effect of temperature on forager abundances varied between sites: temperature was significantly and positively associated with forager abundance at the southern site (Duke Forest), but not at the northern site (Harvard Forest), indicating the importance of geographic location in determining the impact of warming on communities. The effects of geographic location are similarly important among populations of spiders. Spiders from warmer locations are more heat-tolerant than those from cooler locations, leading to variability in foraging behavior (Barton 2011). In ants at least, such geographic variation in response to temperatures likely scales up to influence geographic distributions of species in a warmed world. For instance, Fitzpatrick et al. (2011) suggested that ant assemblages from cooler regions of North America are likely to be more resilient to climatic warming than are assemblages from warmer regions, due, in part, to the smaller range sizes of southern populations.

Furthermore, we found that the effects of warming on species richness varied between sites: species richness at baits was positively correlated with warmer temperatures (Δ°C) at the southern site, but not at the northern site. This increase in richness at the southern site may be the result of combined effects in increased colonization of sites by thermophilic species along with an increase in worker abundance and activity by species already present. Although the overall trend at the southern site was an increase in richness with increasing temperatures, the abundances of some species declined with warming, whereas the abundance of others increased (Pelini et al. 2011a). In particular, foraging by two species (*C. pennsylvanicus* and *A. rudis*) did not depend on temperature, foraging by two others (*F. pallidefulva* and *C. lineolata*) increased with temperature, and foraging by one (*P. imparis*) decreased with temperature. Notably, the two species that responded positively to temperature (*F. pallidefulva* and *C. lineolata*) were observed only at the southern site in this study. However, this was also true of the only species found to respond negatively to warming (*P. imparis,* called the “winter ant” because of its propensity to forage in cooler seasons), which was observed only at Duke Forest and only during the fall sampling event. These species-specific responses are congruent with what we know about the annual and diurnal foraging patterns in these species. Foraging by *P. imparis* is thought to be driven primarily by temperature with an optimal temperature range between 7 and 16°C (Talbot 1943). *Formica pallidefulva* (formerly *F. incerta*) forages almost exclusively during the day, a habit that Talbot (1946) found to be driven by temperature. Talbot never found *F. pallidefulva* foraging below 15.5°C, and suggested that its optimal foraging temperature ranged from 29.5°C and 35°C, although she also observed the species foraging at temperatures exceeding 37.5°C. Likewise, *C. lineolata* responds positively with increased temperature, occurring at higher densities under experimentally warmed conditions (Pelini et al. 2011a). Such variability in responses to warming among ants may lead to shifts in the biogeographic distributions of ant functional traits, although, inversely, this variability in thermal preferences could also result from differences in biogeographic distributions (Fitzpatrick et al. 2011).

Thermal physiology was a strong predictor of the foraging responses of species to experimental climatic warming at the southern site. Species with higher thermal tolerances exhibited more foraging activity in warmer chambers at Duke Forest, and this pattern held during both the summer and fall. This disparate response to temperature across species points to the potential utility of CT_max_ in determining species-specific foraging responses to warming. Indeed, CT_max_ is a strong predictor of cumulative ant activity density in this system: species with higher CT_max_ are generally more abundant in pitfall traps (Diamond et al. 2012b). Unfortunately, there were not enough actively foraging species collected in this study to conduct similar analyses for the Harvard Forest site. However, we note that CT_max_ values for ectotherms tend to be fairly constant across latitudes, likely placing the environmental conditions experienced by northern populations farther from their upper thermal limits (Sunday et al. 2011; Hoffmann et al. 2012). Our results suggest that understanding a key physiological trait can illuminate species-specific responses to climatic warming and potentially lead to the development of robust predictions about the response of biodiversity to warming. In general, there is growing evidence that suggests an important role for physiological traits in better informing species' responses to global climatic warming (Kingsolver 2009; Barton 2011), and our study provides additional support for this relationship. Importantly, our study also establishes a critical link between thermal physiology and species-specific responses to large-scale experimental climatic warming.

These changes in forager abundance did not arise because of variation in recruitment ability with warming because, if a species found a bait, recruitment was essentially equivalent across all temperatures. It is also important to note that the CT_max_ of a species and its abundance in chambers tracked temperature in both the summer, as well as the fall when temperatures were far from CT_max_. This suggests that the relationship between CT_max_ and forager abundance across temperature manipulations may be driven by overall shifts in forager abundances in the warmed chambers rather than behavioral shifts in recruitment ability related to temperature. If this were the case, we would expect to see both altered recruitment ability as a function of temperature as well as a reduced importance of CT_max_ in driving foraging patterns in the warmed chambers when temperature conditions are far from CT_max_. However, we did not collect data on colony size or densities, making it difficult to ascertain the mechanism leading to shifts in forager abundances.

The experimental warming chambers in which this study was conducted are completely open at the top and elevated approximately 2.5 cm from the soil surface at the bottom, meaning that it is possible for the ants we see foraging in the chambers to come from colonies that are outside the chambers (Moise and Henry 2010). While we cannot be certain where the ants are nesting in this study, if the ants we see in the chambers actually came from outside of the chambers, our results still demonstrate that foraging activity depends on temperature. However, we suspect that it is unlikely that many of the ants we see foraging in the chambers come from colonies outside the chambers because the foraging ranges of the most commonly observed species are generally <1 m (Pudlo et al. 1980; personal observation, Lubertazzi 2012). Furthermore, in a pilot experiment, baits stocked with crumbs of Pecan Sandies were placed in each chamber. Out of 72 observations of *Aphaenogaster* spp. foragers returning to a nest with a crumb from the bait, in only three instances was the nest outside the chamber (L. Nichols, unpubl. data). It is possible, however, that some *Camponotus* and *Formica* species may forage many meters (personal observation). But whatever the case, our results stand: these manipulations demonstrate a relationship between experimentally manipulated temperature and foraging activity in ants, regardless of how far ants forage away from their nests.

When temperature altered foraging activity, its effects tended to be present at only the southern site (Duke Forest). This is consistent with the findings of Pelini et al. (2011a) who also showed warming to have less of an impact on species richness and foraging activity at Harvard Forest than Duke Forest. However, it is notable that this trend remains despite the greater magnitude of warming in this study (up to +5°C) relative to that in the Pelini et al. (2011a) study (+1°C, which was conducted with passive warming chambers). The lack of an effect of warming at the northern site may point to the resilience of ants at the northern end of their range to fairly substantial warming. More generally, the results of both the Pelini et al. (2011a) study and ours are consistent with other reports suggesting that warming may be more detrimental at lower latitudes (Deutsch et al. 2008; Tewksbury et al. 2008; Kingsolver 2009; Huey et al. 2012). While these studies typically involve comparisons of tropical and nontropical regions, our study detected significant site-level differences in responses to warming when comparing communities along a latitudinal gradient entirely within the temperate zone. This may suggest that, along with tropical regions, the flora and fauna of low-latitude portions of the temperate zone may also be at increased risk as a result of climate change.

Our study focuses on short-term responses to warming. However, over more generations, the ant populations in our experimental arrays might also adapt to chronic warming (Davis et al. 2005; Skelly et al. 2007; Hof et al. 2011; Hoffmann and Sugò 2011). While the genetic architecture of thermal preference and performance generally remains unknown, recent studies suggests that adaptation – particularly via foraging responses – may be constrained in ectotherms such that they will not be able to evolve fast enough to cope with climatic warming (Davis et al. 2005; Sinervo et al. 2010). Thus, we might expect that, in our experimental warming array, ant species that have a low CT_max_ will not experience sufficient evolutionary change to be able to inhabit the warmest chambers. If indeed limited adaptive ability is a widespread pattern, ants and ectotherms more generally will be reliant on acclimation, behavior, and dispersal responses to warming, i.e. the shorter term types of responses captured by the warming experiment described here. Additionally, shifts in the abundance of foragers on baits are likely to influence reproductive success and survival of colonies, potentially amplifying the impacts of warming on the ant community over time.

In summary, under experimentally warmed conditions, we found that warming altered ant foraging activity, but had a greater impact at the southern range limit than at the northern range limit, and, at least at the southern site, species with higher CT_max_ foraged more heavily at warmer temperatures than did species with lower CT_max_. The altered levels of foraging activity as a result of warming may have important implications for both species persistence as well as ecosystem functioning. Taken together, our results indicate that future research interested in predicting the effects of temperature on the structure and dynamics of communities should consider the behavior and thermal physiology of individual taxa, and how the responses of those taxa vary geographically.
